# Editor-in-Chief’s Note—Thank you to Reviewers

**DOI:** 10.1093/carcin/bgac096

**Published:** 2023-04-22

The Editors would like to thank those listed below who have given their valuable help over the last year, between 1 October 2021 and 30 September 2022, in the review of manuscripts submitted to *Carcinogenesis.*

Francesco Acquati

Rajesh Agarwal

Nobuyoshi Akimitsu

Safiye Aktas

Stefan Ambs

Claudia Andl

Minoti Apte

Margaritis Avgeris

Niranjan Awasthi

Abul Kalam Azad

Narayanasamy Badrinath

Martin Bakht

Allan Balmain

Mary Barcellos-Hoff

Sujit Basu

Frederick Beland

Francesco Bertolini

Vikas Bhardwaj

William Blaner

Laszlo G. Boros

Maarten Bosland

Simon Bouffler

Olivier Boutaud

Chiara Braconi

Frank Braun

Antonino Bruno

Mabel Brunotto

Thomas Burke

Massimiliano Cadamuro

Qiuyin Cai

Shang Cai

Diego Calvisi

Michele Caraglia

Darko Castven

Juan C. Celedón

Wan Chan

Anupam Chatterjee

Ben-Kuen Chen

Fangyao Chen

Yash Choksi

Chaeuk Chung

Mitchell Cohen

Tomer Cooks

Jon Corton

Rutao Cui

Zhi Dai

Kamal Datta

Steven de Jong

Assunta De Rienzo

Niels de Wind

Giannino Del Sal

Cátia Domingues

Lukas Dow

Anna Dubrovska

Hiromichi Ebi

Karam El-Bayoumy

Maria Elola

Isabel Fabregat

Y. Fan

Jing-Xin Feng

Tanis Fenton

Martin Fernandez-Zapico

Fabrizio Fontana

Orazio Fortunato

Peter Friedl

Eitan Friedman

Kazutoshi Fujita

Edward Gabrielson

Cecilia Galvan

David Geller

Kulsoom Ghias

Adam Glick

Noriko Gotoh

Tim Greten

Tom Grimsrud

Michael Grusch

Dongqing Gu

Jian Gu

Yutaka Hashimoto

Naoko Hattori

Stephen Hecht

Tom Hei

Christopher Heinen

Anton Henssen

Tohru Hirota

Izumi Horikawa

Jiajie Hou

Yue-Qing Hu

Xiaoyi Huang

Kay Huebner

Tatsuhiko Imaoka

J. Iwata

Ming-Shiou Jan

Brendan J. Jenkins

Bruno Jham

Deshui Jia

Guangfu Jin

Peter Johansson

Yoshikaz Johmura

Jin-On Jung

Kapil Juvale

Yae Kanai

Min Kang

Shao-Hsuan Kao

Soumendra Karmahapatra

Motohiro Kato

Tom Kelly

Thomas Kensler

Farhan Ullah Khan

Arun Khattri

A. Kobayashi

J. Koentzer

Takashi Kohno

Daizo Koinuma

Yutaka Kondo

Ah-Ng Kong

Xiangyang Kong

Janel Kopp

Achim Krüger

Susan Krum

Donald Kufe

Addanki Kumar

Prabhat Kumar

Kousik Kundu

Satoru Kyo

Maode Lai

Ashish Lal

Tobias Lange

Jeffrey Laskin

Alan Yueh-Luen Lee

Dung-Fang Lee

Ju-Seog Lee

S.C. Lee

Marc Lenburg

Chunying Li

Fang Li

Fuyang Li

Guojun Li

Haitao Li

Hengyu Li

Qi Li

Triantafillos Liloglou

Yih-Cherng Liou

Bin Liu

Caigang Liu

Zhiqiang Liu

Zhuowei Liu

Adhemar Longatto

Dan Lu

Zhongxin Lu

Jianyuan Luo

Stephanie Ma

Yuan Mao

Benoît Marchand

Jens Marquardt

Pierre Martin-Hirsch

Leah Mechanic

Svenja Meierjohann

Keiji Miyazawa

Terry Moody

Morgan Moorghen

Jordan Morant

Hozumi Motohashi

Patricia Muller

Genta Nagae

Hitoshi Nakagama

Yasuhito Nannya

King Pan Ng

Lihong Nie

Sachiyo Nomura

Douglas Noonan

Abraham Nyska

Shuji Ogino

Tatsuya Ohhata

Eiji Oki

Andrew Olaharski

Aikseng Ooi

Tsuyoshi Osawa

Gemma Owens

Alican Özkan

Bhaumik Patel

Ulrich Pfeffer

David Phillips

Kelly Phillips

Gary Piazza

Renate Pilz

Sharon Pine

Krishna Mohan Poluri

Chiara Porta

Ram Raj Prasad

Yiting Qiao

Chengshi Quan

Jianwen Que

L.G. Quiroz

Ana Raimondi

Chinthalapally Rao

Pradip Raychaudhuri

Ian Reddin

Rolland Reinbold

Bárbara Rivera

Martha Robles-Flores

Boris Rodenak Kladniew

Tiago Rodrigues

Stephanie Roessler

Min Ruan

Marek Rusin

Antonio Russo

Akash Sabarwal

Sudipto Saha

Ryoichi Saito

Eiji Sakai

Jonathan Samet

Aranzazu Sanchez

Arandelovic Sandra

Eric Santoni-Rugiu

Andreas Scorilas

Rodney Scott

Karine Serre

Gautam Sethi

Shabana Shabbeer

Zhengjun Shang

Nitesh Sharma

Tatsuhiro Shibata

Noriaki Shimizu

Keiko Shinjo

Tanri Shiozawa

Yohei Shirakami

Xiang Shu

Sukh Mahendra Singh

Evelien Smits

Antonio Solimando

Tom Spratt

Douglas Stairs

Ava Stark

Gary Stoner

Sabrina Strano

Hiromu Suzuki

Akiko Takahashi

Haruhiko Takeda

Kengo Takeuchi

Junko Takita

Shinji Tanaka

Takuji Tanaka

Toshiyasu Taniguchi

Jose Teixeira

Meredith Tennis

Jide Tian

Nikolai Timchenko

Swati Tiwari

Umut Toprak

Darjus Tschaharganeh

Naoto Tsuchiya

Hitoshi Tsuda

Adam Turnbull

Abhishek Tyagi

Geeta Upadhyay

Kiyoe Ura

Angelo Vacca

Darth Vader

Karen Vousden

Chaochen Wang

Chuanxin Wang

Jihan Wang

Jun Wang

Qian Wang

Weilin Wang

Zhe Wang

Fahad Waqar

Masayuki Watanabe

Joseph Wiemels

Vincent Wilson

Hyun Goo Woo

M.-H. Wu

Qingming Wu

Weixiong Xia

Daimin Xiang

Jingwu Xie

Chengguo Xing

Satoshi Yamashita

Chung Yang

Hui Yang

Tao Yang

Xiaobo Yang

Jin-Hai Ye

Lihong Ye

Chi-Tai Yeh

Yao-Tsung Yeh

Kazuhiro Yoshida

Kenichi Yoshida

Rana Youness

Sheng-Jie Yu

W. Yu

Wenqiang Yu

Bi-Feng Yuan

Lixia Yue

Ben Zhang

Lisheng Zhang

Weiying Zhang

Xianglan Zhang

Xing-Ding Zhang

Hua Zhao

Yue Zhao

Jessica Zucman-Rossi

